# Transdiagnostic, Psychodynamic Web-Based Self-Help Intervention Following Inpatient Psychotherapy: Results of a Feasibility Study and Randomized Controlled Trial

**DOI:** 10.2196/mental.7889

**Published:** 2017-10-16

**Authors:** Rüdiger Zwerenz, Jan Becker, Robert Johansson, Ronald J Frederick, Gerhard Andersson, Manfred E Beutel

**Affiliations:** ^1^ Department of Psychosomatic Medicine and Psychotherapy University Medical Center Mainz Johannes Gutenberg University Mainz Mainz Germany; ^2^ Department of Behavioural Sciences and Learning Linköping University Linköping Sweden; ^3^ Center for Courageous Living Los Angeles, CA United States; ^4^ Department of Clinical Neuroscience Centre for Psychiatry Research Karolinska Institutet Stockholm Sweden

**Keywords:** psychoanalytic psychotherapy, emotion-focused therapy, inpatients, aftercare, Internet, clinical trial

## Abstract

**Background:**

Mental disorders have become a major health issue, and a substantial number of afflicted individuals do not get appropriate treatment. Web-based interventions are promising supplementary tools for improving health care for patients with mental disorders, as they can be delivered at low costs and used independently of time and location. Although psychodynamic treatments are used frequently in the face-to-face setting, there has been a paucity of studies on psychodynamic Web-based self-help interventions.

**Objective:**

The objective of this study was to determine the feasibility and preliminary efficacy of a transdiagnostic affect-focused psychodynamic Web-based self-help intervention designed to increase emotional competence of patients with mental disorders.

**Methods:**

A total of 82 psychotherapy inpatients with mixed diagnoses were randomized into two groups. Following discharge, the intervention group (IG) got access to a guided version of the intervention for 10 weeks. After a waiting period of 10 weeks, the wait-list control group (WLCG) got access to an unguided version of the intervention. We reported the assessments at the beginning (T0) and at the end of the intervention, resp. the waiting period (T1). The primary outcome was satisfaction with the treatment at T1. Secondary outcome measures included emotional competence, depression, anxiety, and quality of life. Statistical analyses were performed with descriptive statistics (primary outcome) and analysis of covariance; a repeated measurement analysis of variance was used for the secondary outcomes. Effect sizes were calculated using Cohen *d* and data were analyzed as per protocol, as well as intention-to-treat (ITT).

**Results:**

Patients were chronically ill, diagnosed with multiple diagnoses, most frequently with depression (84%, 58/69), anxiety (68%, 47/69), personality disorder (38%, 26/69), and depersonalization-derealization disorder (22%, 15/69). A majority of the patients (86%, 36/42) logged into the program, of which 86% (31/36) completed the first unit. Satisfaction with the units mastered was rated as good (52%, 16/31) and very good (26%, 9/31). However, there was a steady decline of participation over the course of the program; only 36% of the participants (13/36) participated throughout the trial completing at least 50% of the sessions. According to the ITT analysis, participants improved statistically significantly and with moderate effect sizes (Cohen *d*) compared with the WLCG regarding depression (*d*=0.60), quality of life (*d*=0.53), and emotional competence (*d*=0.49). Effects were considerably stronger for the completers with respect to depression (*d*=1.33), quality of life (*d*=0.83), emotional competence (*d*=0.68), and general anxiety (*d*=0.62).

**Conclusions:**

Although overall program satisfaction and benefit of the program were favorable with respect to the indicators of emotional disorders, the rate of completion was low. Our findings point to the need to target the intervention more specifically to the needs and capabilities of participants and to the context of the intervention.

**Trial Registration:**

Clinicaltrials.gov NCT02671929; https://clinicaltrials.gov/ct2/show/NCT02671929 (Archived by WebCite at http://www.webcitation.org/6ntWg1yWb)

## Introduction

### Background

Mental disorders have become a major health issue worldwide. According to Jacobi et al [1], the 12-month prevalence for mental disorders in Germany was 27.7%, affecting 17.8 million people in the course of 1 year. Despite a broad range of available mental health treatments with established efficacy, less than 50% of individuals afflicted by a mental disorder utilized appropriate health services [1]. Among the reasons for low utilization of mental health care are long waiting times, fear of stigmatization, and time demands of outpatient psychotherapy [2].

Web-based interventions have been considered promising supplementary tools, as they can be delivered at low costs and used independently of time and location [3]. Several meta-analyses have shown medium to high efficacy [4-6], comparable with classical face-to-face therapy [6,7]. In their meta-analyses, Andrews et al [5] concluded that Web-based interventions were well accepted, for example, 86% of participants were satisfied. In a recent study by Richards et al [8], 68% of participants were satisfied with an Web-based intervention for depression.

Web-based self-help interventions have been used in different clinical contexts, for example, self-help for participants with mild to moderate severity of mental complaints [9], combined with outpatient or inpatient psychotherapy [10], or as an aftercare following inpatient treatment [11,12]. In a recent trial by Klein et al [13] with 1013 participants, the effectiveness of a Web-based self-help program for the treatment of mild to moderate depression in different clinical and nonclinical settings could be demonstrated with a between-group effect size of *d*=0.39 post treatment (3 months) and *d*=0.32 in the follow-up (6 months) compared with the usual care (psychological and pharmacological treatments).

Although participants recruited over the Internet fared well, applicability of Web-based self-help interventions for primary care patients has been mixed. Gilbody et al [14] found no benefits of cognitive-behavioral Web-based self-help programs for depression, which had been found efficacious in multiple Web-based trials, compared with the usual care by the general practitioner. Uptake and completion were low despite regular telephone support. While acknowledging potential benefits of the program, participants were struggling with the challenges of their illness, lack of support, and limited personalization of content [15].

In Germany, inpatient psychotherapy is indicated when outpatient psychotherapy has not been sufficient, and severe or multimorbid mental disorders significantly impair activities of daily living and work ability [16]. Although symptoms are expected to improve considerably by inpatient psychotherapy, they may worsen following discharge, particularly when the therapeutic process is not continued by outpatient psychotherapy [17,18].

Although psychodynamic methods are used frequently in outpatient and inpatient treatments in the face-to-face setting [19], the field of Web-based interventions has been dominated so far by cognitive behavioral interventions [6,20,21]. Psychodynamic Web-based interventions have only been developed recently [9,12,22]. Due to the worldwide need for health systems to meet a rising demand for psychotherapeutic treatments, it seems to be logical to take up the challenge from the psychodynamic perspective and develop Web-based interventions, which could improve the shortage [23] of psychotherapeutic treatments.

Efficacy and effectiveness have been proved for psychodynamic psychotherapies for different disorders [24,25], and a recent meta-analysis by Diener et al [26] indicated that facilitation of the affective experience and expression of patients in psychotherapy could further improve treatment results. The affect-focused psychodynamic intervention developed by Johansson et al [27] was based on the affect phobia therapy model by McCullough and Andrews [28] and an adaptation of the concept of mindfulness [29] as outlined in an American self-help book [30]. The affect phobia model postulates that people have become fearful of their feelings, as these had been discouraged, invalidated, or ridiculed by significant persons earlier in their lives [28]. On the basis of the biographical vignettes of patients’ life and treatment experiences, the self-help program guides participants to experience and express their emotions and thus to confront and overcome their maladaptive fears.

Johansson et al [9] recruited participants with depression or anxiety disorders over the Internet. They compared the intervention group (IG) with a control group that received basic support and clinical monitoring of symptoms but no treatment modules or any specific psychotherapeutic support. The between-group effect sizes in the randomized controlled trial (RCT) were moderate (*d*=0.48 for anxiety; *d*=0.77 for depression), and remission rates were significantly higher in the IG compared with the control group.

### Objectives

The primary goal of this study was to test the feasibility of a psychodynamic Web-based self-help intervention for psychosomatic inpatients. For this purpose, we translated and adapted the self-help book *Living Like You Mean It* to the German language and health care system. Unlike Johansson et al [9], we did not recruit via Internet, through advertisement, but rather included participants of inpatient or day clinic treatment before discharge. As facilitation of emotional experience is one of the core processes in psychodynamic psychotherapy [31], we enlarged the scope of the transdiagnostic study to a broad range of mental disorders.

Beyond that, we wanted to gather first hints of efficacy of the intervention regarding emotional competence, depression, and anxiety with an RCT.

### Hypotheses

On the basis of a previous study [9], we hypothesized that at least 75% of the participants of the IG will be “very satisfied” or “mainly satisfied” with the intervention, and that at least 50% of them will complete all 8 units.

Furthermore, we expected the participants of the IG to show significantly higher emotional competence, lower depression, and anxiety scores at the end of the intervention compared with the participants of the control group.

## Methods

### Recruitment

Becker et al [10] had described that inpatients and day clinic patients of the Department of Psychosomatic Medicine and Psychotherapy who were above the age of 18 years and had Internet access and an email address were eligible to participate. Patients with acute suicidality, psychosis, current alcohol or drug addiction, and a lifetime diagnosis of schizophrenia, schizoaffective, bipolar, or organic psychiatric disorder were excluded. Patients were informed about the study and its rationale in an information session during their inpatient or day clinic treatment. After giving written informed consent, eligible patients were coded and randomized by block randomization at a ratio of 1:1 with the help of the computer software Research Randomizer provided in the Web by Urbaniak and Plous [32]. Upon discharge, they received their log-in to the Web-based self-help intervention.

### Intervention

The intervention was based on the self-help book *Living Like You Mean It* by Ronald J Frederick [30], which the Swedish work group around Gerhard Andersson recently adapted in their trial [9,27]. We translated the original English manuscript and adapted the content to the German health care system and culture. We compared our version with the Swedish one, and we translated and revised the tasks [9]. The program was piloted with psychotherapy inpatients and thoroughly revised by experts (2 experienced psychotherapists of our clinic).

In this book, 8 units—corresponding to the chapters—cover four steps: enhancing awareness of one’s emotions and related defenses, regulating the anxiety that emerges when feared emotions are approached, fully experiencing, and mindfully expressing emotions to other people [10]. All units are presented online in a consecutive order, supplemented with questions and tasks for the participants to work through after completion of each unit. Among various exercises, mindfulness was included as text instructions and audio files according to Kabat-Zinn [29]. Attending to bodily felt experience was regarded as a major venue for feeling and regulating emotions.

Upon discharge from inpatient or day clinic treatment, the IG got access to the intervention for 10 weeks when they first logged onto the platform (Multimedia Appendix 1). Participation was free of charge. To complete the intervention within 10 weeks, participants were asked to do one unit (Multimedia Appendix 2) per week. Only when participants had answered all questions in the unit´s tasks (Multimedia Appendix 3) and transmitted them to the Web-based therapist, a unit was considered to have been completed. Encouraging feedback was delivered within 2 weekdays after transmission of their replies by a trained psychologist who was supervised by 2 experienced psychotherapists, familiar with the intervention.

### Control Condition

The study was performed using a wait-list control design to evaluate the efficacy of the intervention developed. Patients in the wait-list control group (WLCG) started their intervention 10 weeks after discharge from inpatient or day hospital treatment, when the intervention of the IG had ended. Additionally, the WLCG received an unguided version of the intervention and therefore did not receive feedback from a Web-based therapist (to be analyzed separately).

### Outcomes

All questionnaires were given online. Assessments were performed at discharge from the clinic (T0), at the end of the intervention of the IG (T1), 2 months later for follow-up assessment (T2; only IG), and at the end of the intervention of the WLCG (T3; only WLCG).

### Primary Endpoint

Satisfaction with the intervention in the IG as the primary endpoint was measured with one item of the German version of the Client Satisfaction Questionnaire (CSQ-8) [33] at T1. The item “How satisfied are you with the Web-based self-help program overall?” was rated on a 4-point Likert scale (“very satisfied,” “mostly satisfied,” “slightly satisfied,” “rather dissatisfied”). We used this item instead of the scale score because it was more appropriate to compare the results with the original Swedish study [9], which reported frequencies of overall satisfaction (82%) with the Web-based self-help intervention on which our program was based.

Additionally, we assessed satisfaction on a weekly basis with the item “Please rate the unit as a whole.” on a 5-point Likert scale (“very good,” “good,” “satisfactory,” “bad,” “very bad”).

Additionally, participants rated their satisfaction with each unit completed on a 5-point Likert scale from “bad” to “very good.”

### Secondary Endpoints

Emotional competence was assessed with the German version of the 27-item Emotion-Regulation Skills Questionnaire (ERSQ) [34]. As items range from 0 to 4, they are summed up to a score from 0 to 108. Internal consistency is high (Cronbach alpha=.90).

The Patient Health Questionnaire-9 (PHQ-9) [35] was used to assess depressive symptoms. Adding the scores of the 9 items (from 0-3), the total score ranges from 0 to 27. Scores below 5 are labeled minimal, scores between 5 and 9 mild, from 10 to 14 as moderate, and above 14 as severe depressive symptoms. Psychometric properties are sound with Cronbach alpha ranging between .86 and .89.

Anxiety was assessed by the General Anxiety Disorder-7 (GAD-7) [36,37], which is based on the same Likert scale and has corresponding cut-offs as the PHQ-9. Its validity has been verified and its Cronbach alpha of .92 demonstrated a sufficient reliability.

Depersonalization was assessed with the 2-item version of the Cambridge Depersonalization Scale (CDS-2) [38] describing the feeling of being detached from one’s body, thoughts, or emotions. The CDS-2 sum score (range 0-6, scoring format is identical with the GAD-2) correlates strongly with clinician rated depersonalization severity (*r*=.77) with a sensitivity of 78.9% and a specificity of 85.7%.

Quality of life was measured with the reliable and valid European Health Interview Survey Quality of Life 8-item index (EUROHIS-QOL-8) [39], a shortened version of the World Health Organization Quality of Life Instrument-Abbreviated Version (WHOQOL-BREF) using a Likert scale ranging from 0 to 4. A higher mean score indicates better quality of life.

The Rosenberg Self-Esteem Scale (RSE) [40] was used to assess self-esteem by 10-Likert scale items ranging from 0 to 3. Higher scores imply higher self-esteem. Internal consistency (Cronbach alpha=.84) and validity have been shown in a previous study [41].

The subjective prognosis of gainful employment (SPE) [42] assessed the subjective prognosis of gainful employment with 3 items, resulting in a score between 0 and 3, so that a higher score indicates a higher risk for work disability or early retirement.

The 8-item Somatic Symptom Scale (SSS-8) [43] is a reliable and valid self-report measure covering gastrointestinal, pain, fatigue, cardiopulmonary, and general somatic symptoms burden over the past 7 days (0 = “not at all” to 4 = “very much”).

Symptoms of depression and anxiety were measured after completing every unit with the PHQ-4 [44]. The PHQ-4 is a very short, reliable, and valid combination of items from the PHQ-9 and the GAD-7, consisting of four items, two for anxiety and depression each.

Completion of units was determined objectively on the basis of entries in the database of the platform.

### Statistical Methods

The primary outcome was evaluated with descriptive statistics. Secondary outcomes were analyzed by analysis of covariance (ANCOVA) controlling outcome variables by their baseline scores and a repeated measurement analysis of variance (ANOVA) for the weekly assessment of the PHQ-4. With the participants’ written consent, diagnoses were taken from the clinical documentation of the Department of Psychosomatic Medicine and Psychotherapy. Effect sizes were calculated transforming the eta-squared from ANCOVA into Cohen *d* to estimate treatment effects controlled for the baseline score. Multiple imputations resulted in implausible results by overestimating effects because of missing data, especially in participants with less completed units. Therefore, last observation carried forward (LOCF) was used as a conservative approach in an intention-to-treat analysis (ITT). All analyses have been conducted with IBM SPSS Statistics version 23 [45].

As this was a feasibility study, no power analysis was conducted. Over the course of 20 weeks (from September 2015 to February 2016), consecutive patients (inpatient and day hospital) treated at the Department of Psychosomatic Medicine and Psychotherapy were invited to participate in the trial. As the clinic treats about 390 inpatients per year and we assumed a participation rate of approximately 30% [11], we expected a sample size of 66 patients.

### Ethics and Data Security

Randomization of participants and storing of personal data were conducted by the Study Center of Mental Disorders at the University Medical Center Mainz. Management of the study, administration of the Internet platform, and therapeutic feedback for the patients in the IG were performed by psychologists of the Department of Psychosomatic Medicine and Psychotherapy. A firewall-protected Web server using a secure sockets layer–encrypted access to the platform itself and the database containing the log-in information hosted the study platform. All questionnaires were administered Web-based with SoSci Survey [46]. Patients logged in on the study platform using pseudonyms. As no personal data were stored on the Web server, personal data of users could not be identified.

The Ethics Committee of the Statutory Physician Board of the State of Rhineland-Palatinate approved the clinical protocol and written informed consent (Ref. No. 837.299.15-10067), and all procedures described in the clinical trial protocol (ClinicalTrials.gov Identifier: NCT02671929) follow the ICH-GCP guidelines and ethical principles described in the current revision of the Declaration of Helsinki. Local legal and regulatory requirements were abided.

## Results

### Study Flow and Patient Characteristics

**Figure 1:**
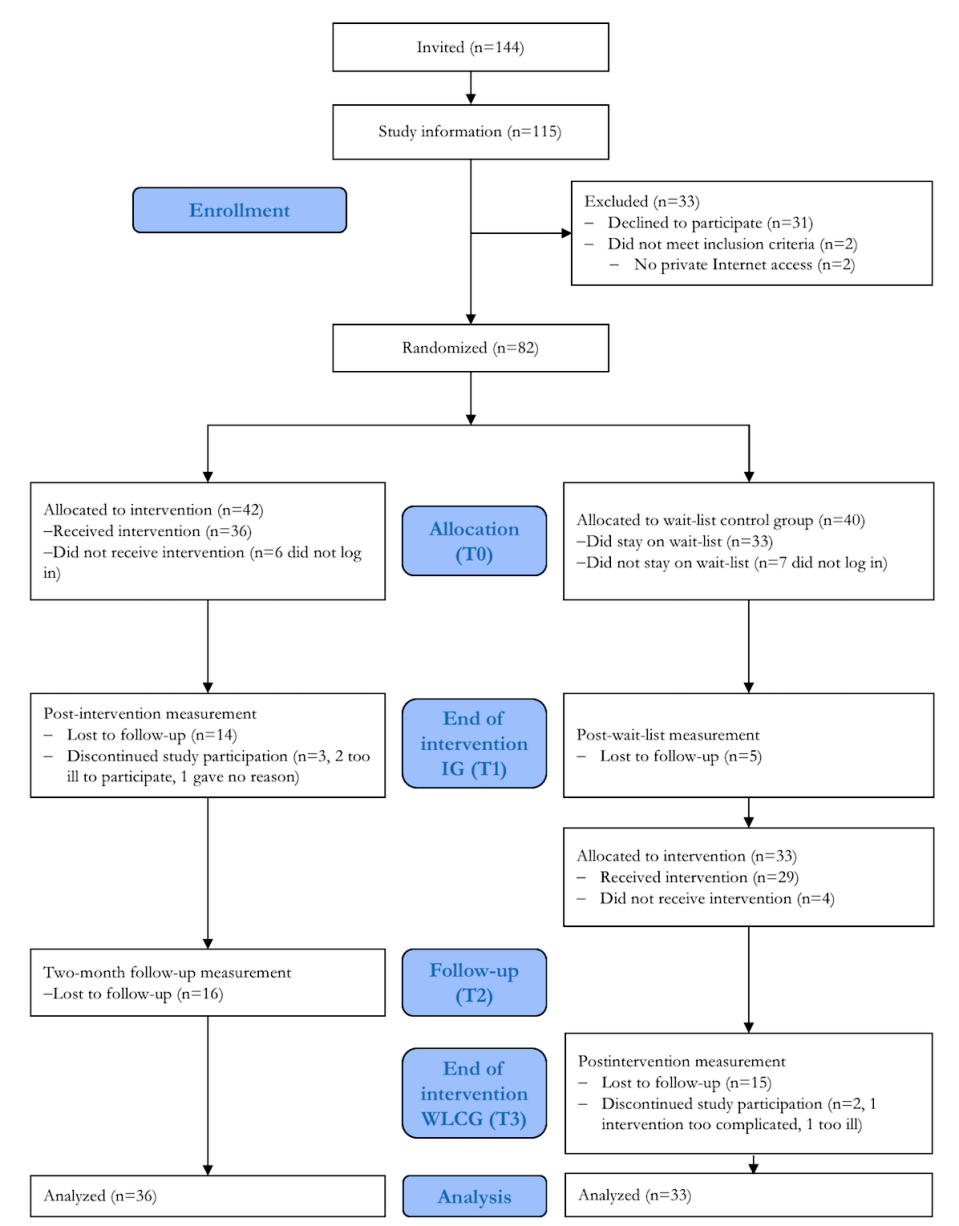
The CONSORT (Consolidated Standards of Reporting Trials) flow diagram.

Figure 1 shows the flow of participants in the study. Out of 144 patients approached, 115 patients participated in the study information session. Of these, 26.9% (31/115) declined to participate. Two patients willing to participate had to be excluded because they had no private Internet access.

A total of 82 participants were randomized to IG or WLCG. In addition, 86% (36/42) of the IG logged into the platform (values for WLCG were similar), and another 86% (31/36) completed the first unit. Nonstarters (IG: n=6; WLCG: n=7) were more often male than female (53.8% vs 46.2%) and more likely to be part-time employed than starters but not on a statistical significant level.

Furthermore, 7 patients dropped out, 4 from the IG and 3 from the WLCG group; the reasons that they named were health problems (n=3), lack of time (n=2), and problems of dealing with the Web-based intervention (n=1), and one patient gave no response. Dropouts were older (mean 50.80, SD 7.92 vs mean 39.06, SD 14.36) than participants (*t*_6,3_=2.96, *P*=.02, *d*=1.37); otherwise, there were no differences. At the end of the treatment phase, waiting period (T1), 61% (50/82) of randomized participants completed the assessment.

Participants who completed the T1 assessment did not differ from those participants who dropped out concerning sociodemographic characteristics and baseline mental symptoms. For all outcome analyses, patients were excluded when the baseline assessment (T0) was missing (n=13). A total of 69 participants were analyzed after substituting in the missing data based on LOCF.

In the IG, 13 participants completed the intervention, that is, logged into the intervention continuously for 10 weeks and finished at least 50% of the units. Table 1 shows the demographic and medical baseline data, separately for the IG and the WLCG.

The majority of participants were female and unmarried; mean age was 40 years (IG: mean 38.92, SD 12.66; WLCG: mean 41.00, SD 16.00). Despite good education, only slightly over half of them were working or in training. Most frequent main diagnoses were affective, anxiety, and personality disorders, followed by depersonalization-derealization disorder and somatoform disorders. Substance abuse, eating disorders, and obsessive-compulsive disorders, etc, were classified as “others” in the table according to the International Statistical Classification of Diseases, Tenth Revision [47]. The majority (83%, 57/69) had more than one diagnosis.

As the baseline data (T0) in Table 1 show slight differences exist between IG and WLCG on entry into the study. The IG had lower scores of depersonalization-derealization symptoms (CDS-2) than the WLCG (ITT: *P*=.02, Completer: *P=*.01). Furthermore, the completer in the IG reported a higher quality of life at baseline than the WLCG (*P*=.04).

### Primary Outcome

The majority was mostly (57%, 12/21) or very satisfied (38%, 8/21); only one participant (5%, 1/21) was slightly dissatisfied (based on the one item of the CSQ-8). Asked whether they would do the Web-based self-help program once more if they needed help, more than half of the participants (57%, 12/21) said “definitely yes,” about one third (29%, 6/21) said, “I believe so,” and only few (14%, 3/21) answered, “I do not believe so.” As overall satisfaction in the CSQ-8 at T1 was only rated by a small proportion of participants who had completed assessments at termination (n=21), we also analyzed ratings of the quality of the units mastered in the Web-based self-help program. For this purpose, we used the ratings for the last session completed by each participant. On the basis of total 31 responses, satisfaction was judged as very good (29%, 9/31), good (52%, 16/31), satisfactory (16%, 5/31), and bad (3%, 1/31). Thus, overall satisfaction exceeded our expectations of 75%.

The total score of the CSQ-8 in the IG (mean 26.33, SD 2.89) was above the cut-off (24.5), which indicates a high treatment satisfaction as calculated in a large sample of inpatients of psychosomatic rehabilitation [48].

### Secondary Outcomes

Table 2 shows descriptive statistics for secondary outcomes, and Table 3 presents the test statistics.

Tables 2 and 3 show that the IG improved regarding the secondary outcome criteria, whereas the WLCG deteriorated. Compared with the WLCG, there were significant benefits in the IG regarding depressive symptoms, quality of life, and a trend to improvement regarding emotional competence in the ITT analyses (LOCF). Effect size differences were in the moderate range. Among completers of the intervention, there were significant improvements regarding emotional competence, depression, anxiety, and quality of life. Effect size differences were large (depression, quality of life) to moderate.

Psychological complaints in the course of the intervention assessed with the PHQ-4 did not change over time in the IG using a repeated measurement ANOVA with LOCF (*F*_7,210_=0.66; *P*=.70).

**Table 1:** Demographic and medical data of study participants.

Demographic and medical data		IG^a^ (n=36), n (%)	WLCG^b^ (n=33), n (%)	Total (N=69)^c^, n (%)
**Sex**				
	Female	29 (81)	20 (61)	49 (71)
	Male	7 (19)	13 (39)	20 (29)
**Marital status**				
	Single	23 (64)	19 (61)	42 (63)
	Married	8 (22)	9 (29)	17 (25)
	Separated, divorced, widowed	5 (14)	3 (10)	8 (12)
**Education**				
	No, lower, or other graduation	6 (17)	1 (4)	7 (11)
	Middle secondary	9 (25)	16 (51)	25 (37)
	Higher secondary	21 (58)	14 (45)	35 (52)
**Employment**				
	Full-time	13 (36)	7 (21)	20 (29)
	Part-time	7 (19)	4 (12)	11 (16)
	Apprenticeship	4 (11)	1 (3)	5 (7)
	Retired	3 (8)	3 (9)	6 (9)
	Not working	3 (8)	15 (46)	18 (26)
	Other	6 (17)	3 (9)	9 (13)
**Medical diagnoses^d^**				
	Affective disorders (F30-F34)	27 (75)	31 (94)	58 (84)
	Anxiety disorders (F40-F41)	21 (58)	26 (79)	47 (68)
	Personality disorders (F60-F69)	12 (33)	14 (42)	26 (38)
	Depersonalization-derealization disorder (F48.1)^e^	4 (11)	11 (33)	15 (22)
	Somatoform (F45)	3 (8)	3 (9)	6 (9)
	Others (including F1x; F42; F50)	18 (50)	14 (42)	32 (46)
**Ongoing outpatient psychotherapy at intake**				
	Yes	13 (36)	12 (39)	25 (37)
	No	23 (64)	18 (58)	41 (61)

^a^IG: intervention group.

^b^WLCG: wait-list control group.

^c^With the exception of gender, data on 2 persons missing.

^d^Multiple diagnoses; International Statistical Classification of Diseases (ICD-10) codes in parenthesis.

^e^Significant difference between groups; χ^2^_1_=4.7, *P*=.03, Cramer *V*=.26.

**Table 2:** Descriptive statistics of outcome criteria at baseline and at the end of the intervention: intervention group (total, completers) versus wait-list control group.

Outcomes	T0^a^			T1^b^		
	IG_total_^c^ (n=36), mean (SD)^d^	IG_comp_ (n=13)^e^, mean (SD)	WLCG_total_^f^ (n=33), mean (SD)	IG_total_ (n=36), mean (SD)	IG_comp_ (n=13), mean (SD)	WLCG_total_ (n=33), mean (SD)
Emotion Regulation Skills Questionnaire (ERSQ)	61.75 (17.13)	66.85 (8.58)	60.09 (15.22)	63.84 (18.24)	69.92 (13.85)	56.24 (15.60)
Patient Health Questionnaire 9 (PHQ-9)	11.92 (5.46)	10.08 (4.92)	12.06 (5.7)	11.06 (6.49)	7.23 (3.92)	13.15 (5.89)
Generalized Anxiety Disorder Assessment (GAD-7)	10.53 (4.9)	9.46 (5.17)	10.61 (5.15)	10.11 (5.42)	7.23 (4.13)	10.30 (5.25)
European Health Interview Survey Quality of Life 8 (EUROHIS-QOL-8)	2.04 (0.69)	2.38 (0.47)	1.98 (0.62)	2.15 (0.88)	2.65 (0.73)	1.87 (0.66)
Rosenberg Self-Esteem Scale (RSE)	18.83 (7.31)	22.46 (5.30)	20.42 (6.45)	17.08 (8.33)	19.61 (7.02)	17.39 (6.92)
Somatic Symptom Scale (SSS-8)	11.42 (5.54)	8.92 (4.46)	11.76 (6.65)	11.06 (6.13)	8.69 (5.28)	12.64 (6.73)
Cambridge Depersonalization Scale Short Version (CDS-2)	1.47 (1.89)	1.15 (1.46)	2.76 (2.61)	1.31 (1.69)	0.69 (0.95)	2.55 (2.39)
Subjective prognosis of gainful employment (SPE)	1.36 (1.27)	0.69 (1.03)	1.15 (1.18)	1.17 (1.21)	0.46 (0.97)	1.03 (1.10)

^a^T0: Baseline.

^b^T1: End of intervention.

^c^IG: intervention group.

^d^SD: standard deviation.

^e^Completers of the IG (10 weeks log-in and >50% of the units completed) *.*

^f^WLCG: wait-list control group.

**Table 3:** Test statistics: intention-to-treat (ITT) and completer analyses comparing intervention group (ITT, completers) and wait-list control group.

Outcomes	T0^a^				T1^b^					
	ITT^c^		Completer		ITT			Completer		
	*t*_67_	*P*	*t*_44_	*P*	*F*_1,66_	*P*	*d*	*F*_1,43_	*P*	*d*
Emotion Regulation Skills Questionnaire (ERSQ)	0.42	.67	1.50	.14	3.89	.05	0.49	4.98	.03	0.68
Patient Health Questionnaire-9 (PHQ-9)	0.11	.92	1.10	.28	6.01	.02	0.60	19.01	<.001	1.33
Generalized Anxiety Disorder Assessment (GAD-7)	0.07	.95	0.68	.50	0.02	.88	0.00	4.09	.049	0.62
European Health Interview Survey Quality of Life 8 (EUROHIS-QOL-8)	0.38	.70	2.12	.04	4.63	.04	0.53	7.35	.01	0.83
Rosenberg Self-Esteem Scale (RSE)	0.96	.34	1.01	.32	0.81	.37	0.22	0.12	.73	0.11
Somatic Symptom Scale (SSS-8)	0.23	.82	1.41	.17	2.41	.01	0.38	1.51	.23	0.38
Cambridge Depersonalization Scale Short Version (CDS-2)	2.33	.02	2.63	.01	0.70	.41	0.21	2.03	.08	0.55
Subjective prognosis of gainful employment (SPE)	0.71	.48	1.23	.23	0.15	.70	0.09	1.60	.21	0.39

^a^T0: baseline; *t* test with test statistics (*t*_df_), level of significance (*P*).

^b^T1: End of intervention; analysis of covariance with *F* test statistics (*F*_df_), level of significance (*P*) and effect size (Cohen *d*).

^c^ITT: Intention-to-treat.

## Discussion

### Principal Findings

Web-based self-help interventions have proven effective in treating different kinds of mental disorders across a broad range of health contexts as single intervention as well as supplements to face-to-face treatments. However, although about half of face-to-face interventions in Germany have been psychodynamic, there has been little research on psychodynamic Web-based self-help. We have chosen affect-focused psychodynamic psychotherapy, which has proven an effective Web-based treatment for anxiety and depression [9]. As the original trial yielded promising results on the basis of the patients with generalized anxiety disorder recruited over the Internet, we decided to test the feasibility and efficacy of psychodynamic Web-based self-help for a wider range of patients from clinical practice. We proceeded similarly to Farchione et al [49], who studied a unified protocol for a transdiagnostic emotion-focused cognitive behavioral therapy for emotional disorders. Similar to Moses and Barlow [50], we presumed that avoidance of emotional experience is a widespread factor in mental disorders [31], and we therefore enlarged the scope of the transdiagnostic study to a broad range of mental disorders. On the basis of the previous findings [9], we had expected improvement of depression, anxiety, and emotional competence.

In addition, our purpose was shifted from a single intervention to aftercare following inpatient or day clinic psychotherapy. Keeping these substantial differences to the previous trial in mind, our primary aim was to investigate the feasibility of our intervention.

Acceptance was good, that is, only 26.9% (31/115) of those attending the information session declined participation. Of those randomized to the IG, the great majority (86%, 36/42) logged into the program, and of these, 86% (31/36) completed the first unit (similar values were found for the WLCG. Satisfaction was rated highly at 95% (20/21). Due to the high proportion of missing data at the follow-up assessment, we additionally used the ratings for individual sessions completed, which still met our expectations of at least 75% satisfied, that is, rated as good (52%, 16/31) and very good (29%, 9/31). However, there was a steady decline of participation from unit 1 to unit 8; only 13/36 participants (36%) were actively participating throughout the entire program, completing at least 50% of the units. Similar to a trial by Gilbody et al [14,15], the major reasons for dropping out of the program were that it was considered too demanding and exhausting. Indeed, participants reported spending an average of 5 hours per week with the program, which can be considered a barrier to completion.

However, results pointed to considerable benefit from participation in the program. According to the conservative estimates of ITT analysis with LOCF, participants improved significantly and with moderate effect sizes compared with the WLCG with improvements in depression, quality of life, and emotional competence (trend). Effects of the small number of completers were considerably stronger. In line with previous findings [9], they reached strong effects regarding depression and anxiety and moderate effects regarding emotional processing.

Although we had proposed that Web-based self-help may fill a gap between inpatient treatment and aftercare, we had not anticipated that 82% (18/22) in the IG and 68% (18/27) in the WLCG continued previous psychotherapy or started a new face-to-face treatment during our trial. We cannot be sure whether continuing or starting psychotherapy may have further discouraged participation in the Web-based self-help program because of additional time demands, and we cannot tell whether psychotherapists were informed of program participation by their patients and whether they were encouraging or discouraging toward participation by their patients.

### Limitations

There was a striking discrepancy between overall program satisfaction and benefit and the low rate of completion of the program, which we had not anticipated. This stands in contrast to the previous trial by Johansson et al [9], where 84% of patients with anxiety or depressive disorders completed all modules, receiving comparable encouragement by a therapist. Although Johansson et al [9] had recruited participants over the Internet, we recruited patients who had just undergone lengthy and intensive psychotherapy on an inpatient or day hospital basis. Comparisons between trials need to be cautioned by the greater diagnostic heterogeneity, which we had sought by purpose. Clearly, the participants in the study of Johansson et al [9] had a higher symptom load at the start than our group, which may have motivated patients to follow through with the program and limited the gains, which could be achieved in our trial.

A strength of this study was that we applied it in a clinical setting, recruiting patients from mental health care treatment. However, following intensive inpatient psychotherapy, our effect sizes can be expected to be smaller compared with trials when patients are recruited for primary Web-based treatment. Our findings alert us to the significance of the context of additional mental health treatments. An ongoing psychotherapy may have further diminished motivation and time for Web-based treatment, which was present in a substantial part of our IG but almost absent in the Swedish group. Although both groups resembled each other in terms of age, gender, and education, unlike the Swedish participants, our patient group was predominantly single and a high percentage was not working. From our clinical point of view, we would see this as being indicative of a chronically sick sample, with a considerable comorbidity of personality disorders, whose difficulties in life adjustment may have well impeded the ability to follow through a Web-based program that requires substantial self-directed effort.

As this was a feasibility study, the sample was small, particularly in the completer group. Therefore, we cannot differentiate compliance and success between subgroups (eg, the presence or absence of personality disorder, the structure of work, or outpatient psychotherapy).

Unfortunately, it is also not possible to compare the effects at the end of the intervention between the IG and the WLCG because participants of the WLCG only got access to the intervention after a waiting period of 10 weeks.

### Conclusions

Our findings point to the requirement to target the intervention more specifically to the needs and capabilities of participants and the context of traditional mental health care. Although participants were satisfied with our intervention and gained significant benefits, the majority was not willing or able to follow it through to completion. To reduce the considerable weekly time demands, we have increased flexibility of participation differentiating between the required and optional exercises and extended the time allotted. To increase familiarity and compliance under less challenging conditions, we plan to offer participation to future patients routinely during inpatient treatment—given positive experiences in a recent trial combining deprexis24 with inpatient psychotherapy [51]. We believe that initiating the program in a structured and supportive therapeutic setting may make it easier to continue program participation when participants are on their own during aftercare.

## Appendix

Multimedia Appendix 1 Screenshot of the Home page.

Multimedia Appendix 2 Screenshot of unit 2 of the intervention.

Multimedia Appendix 3 Screenshot of task 1 from unit 2 of the intervention.

Multimedia Appendix 4 CONSORT EHEALTH form V1.6.

## Abbreviations

ANCOVA: analysis of covariance
ANOVA: analysis of variance
CDS-2: Cambridge Depersonalization Scale
CSQ-8: Client Satisfaction Questionnaire
ERSQ: Emotion Regulation Skills Questionnaire
EUROHIS-QOL-8: European Health Interview Survey Quality of Life 8-item index
GAD-7: General Anxiety Disorder Screener
IG: intervention group
ITT: intention-to-treat
LOCF: last observation carried forward
PHQ-9: Patient Health Questionnaire
RCT: randomized controlled trial
RSE: Rosenberg Self-Esteem Scale
SPE: Subjective prognosis of gainful employment scale
SSS-8: Somatic Symptom Scale
WLCG: wait-list control group
