# Supplementary figures and images for: Transdiagnostic, Psychodynamic Web-Based Self-Help Intervention Following Inpatient Psychotherapy: Results of a Feasibility Study and Randomized Controlled Trial
Source: JMIR Ment Health. 2017 Oct 16;4(4):e41. doi: 10.2196/mental.7889 (PMC5662790; doi:10.2196/mental.7889)

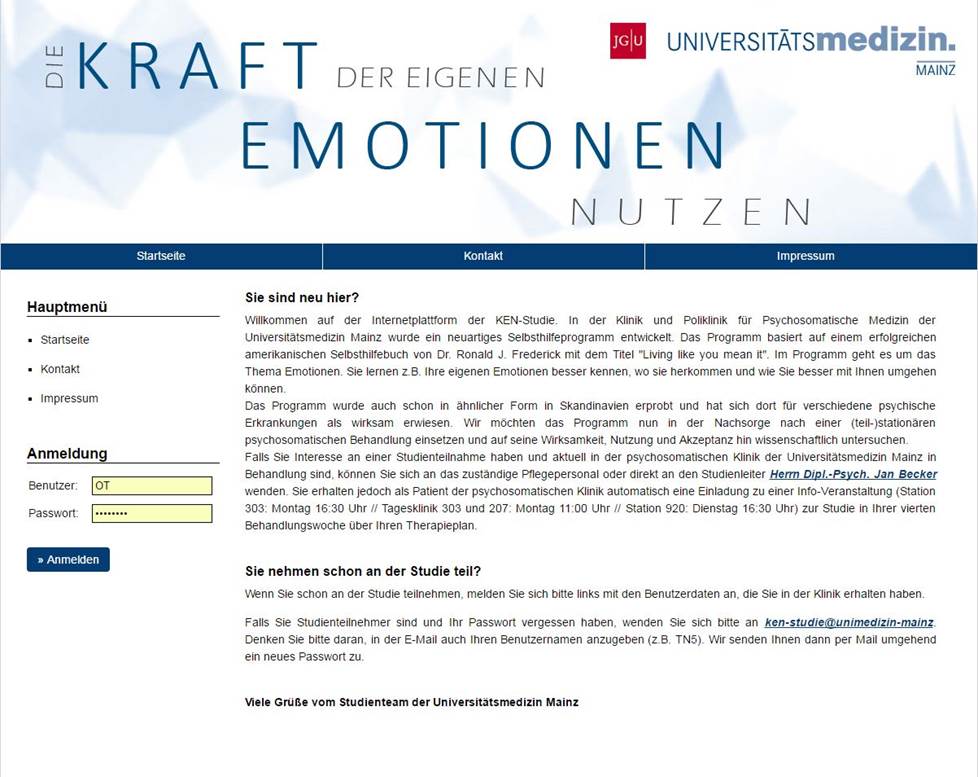

Supplement: Multimedia Appendix 1 [file mental_v4i4e41_app1.jpg]

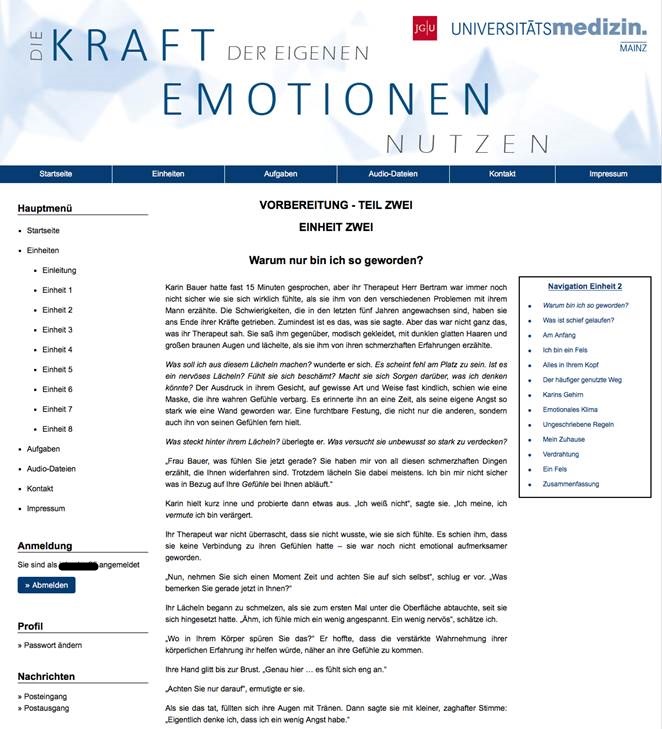

Supplement: Multimedia Appendix 2 [file mental_v4i4e41_app2.jpg]

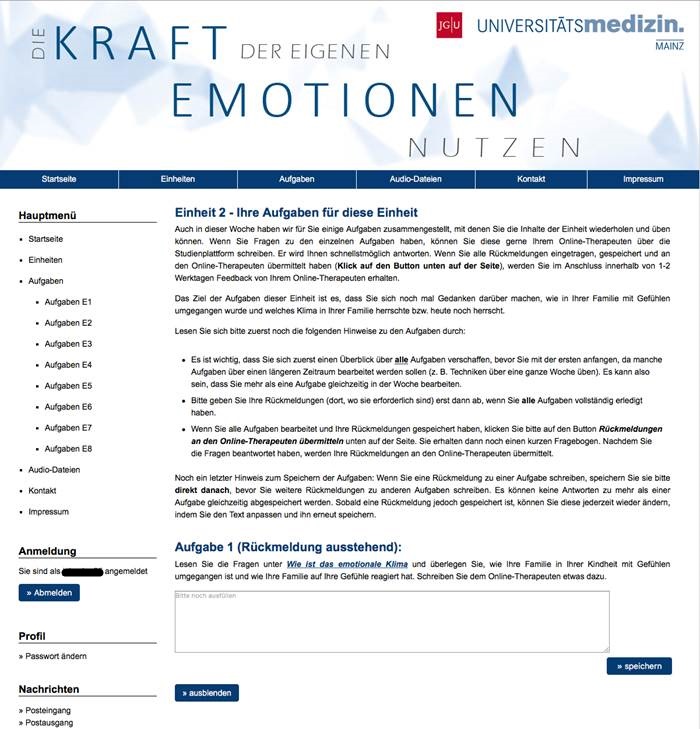

Supplement: Multimedia Appendix 3 [file mental_v4i4e41_app3.jpg]
